# Sustainable Intensification of Beef Production in the Tropics: The Role of Genetically Improving Sexual Precocity of Heifers

**DOI:** 10.3390/ani12020174

**Published:** 2022-01-12

**Authors:** Gerardo Alves Fernandes Júnior, Delvan Alves Silva, Lucio Flavio Macedo Mota, Thaise Pinto de Melo, Larissa Fernanda Simielli Fonseca, Danielly Beraldo dos Santos Silva, Roberto Carvalheiro, Lucia Galvão Albuquerque

**Affiliations:** 1 Animal Science Department, School of Agricultural and Veterinary Sciences, São Paulo State University (UNESP), Jaboticabal 14884-900, SP, Brazil; delvanzsilva@gmail.com (D.A.S.); lsimielli.fonseca@gmail.com (L.F.S.F.); daniellyberaldo@gmail.com (D.B.d.S.S.); roberto.carvalheiro@unesp.br (R.C.); 2 Department of Agronomy, Food, Natural Resources, Animals and Environment (DAFNAE), University of Padova, Viale dell’ Università 16, 35020 Legnaro, Italy; flaviommota.zoo@gmail.com; 3 Animal Science Department, Federal University of Santa Maria, Santa Maria 97105-900, RS, Brazil; thaise_p.melo@hotmail.com; 4 Animal Science Department, José do Rosário Vellano University (UNIFENAS), Alfenas 37132-440, MG, Brazil; 5 National Council for Scientific and Technological Development (CNPq), Brasília 71605-170, DF, Brazil

**Keywords:** beef cattle, early pregnancy, genomic selection

## Abstract

**Simple Summary:**

Tropical pasture-based beef production systems play a vital role in global food security. The importance of promoting sustainable intensification of such systems has been debated worldwide. Demand for beef is growing together with concerns over the impact of its production on the environment. Implementing sustainable livestock intensification programs relies on animal genetic improvement. In tropical areas, the lack of sexual precocity is a bottleneck for cattle efficiency, directly impacting the sustainability of production systems. In the present review we present and discuss the state of the art of genetic evaluation for sexual precocity in *Bos indicus* beef cattle, covering the definition of measurable traits, genetic parameter estimates, genomic analyses, and a case study of selection for sexual precocity in Nellore breeding programs.

**Abstract:**

Increasing productivity through continued animal genetic improvement is a crucial part of implementing sustainable livestock intensification programs. In Zebu cattle, the lack of sexual precocity is one of the main obstacles to improving beef production efficiency. Puberty-related traits are complex, but large-scale data sets from different “omics” have provided information on specific genes and biological processes with major effects on the expression of such traits, which can greatly increase animal genetic evaluation. In addition, genetic parameter estimates and genomic predictions involving sexual precocity indicator traits and productive, reproductive, and feed-efficiency related traits highlighted the feasibility and importance of direct selection for anticipating heifer reproductive life. Indeed, the case study of selection for sexual precocity in Nellore breeding programs presented here show that, in 12 years of selection for female early precocity and improved management practices, the phenotypic means of age at first calving showed a strong decreasing trend, changing from nearly 34 to less than 28 months, with a genetic trend of almost −2 days/year. In this period, the percentage of early pregnancy in the herds changed from around 10% to more than 60%, showing that the genetic improvement of heifer’s sexual precocity allows optimizing the productive cycle by reducing the number of unproductive animals in the herd. It has a direct impact on sustainability by better use of resources. Genomic selection breeding programs accounting for genotype by environment interaction represent promising tools for accelerating genetic progress for sexual precocity in tropical beef cattle.

## 1. Introduction

Tropically adapted cattle under a grass-fed system characterizes the beef production in the tropics [1]. Relying on the use of pastures, beef produced in tropical areas plays an important role in global food security. It is a rich protein source to humans from inedible resources and, because of this, contributes to reducing the demand and prices for grains around the world [2]. However, tropical beef production intensification, if not sustainable, could result in extensive negative environmental impacts by tending to expand into natural ecosystems [3,4]. Basically, sustainable intensification interlinks food security and environmental protection by focusing on the increase of productivity from existing farmlands without further land conversion to agriculture or loss of natural ecosystems [4]. 

To be sustainable, cattle production systems must attend to the farmer, industry, and society requirements, which include cost-effectiveness, high production, animal welfare, and minimal environmental pollution [5]. Indeed, livestock plays an important role in employment and rural economies but is constantly related to environmental concerns, including greenhouse gas emissions and deforestation [4]. In this sense, sustainable livestock intensification programs appeared as an alternative to produce more output per area through more efficient use of resources in order to achieve production systems that are profitable, socio-culturally acceptable, beneficial to the people, and protective of natural resources [6]. 

An effective way to increase productivity is through continued animal genetic improvement, making it a crucial part of implementing sustainable livestock intensification programs [6]. Selecting for heifer sexual precocity, for example, may increase the overall cow’s productivity and profitability. It contributes to anticipating female productive life and is favorably genetically associated with lifetime reproductive performance in tropically-adapted beef cattle [7,8,9]. In addition, sexually precocious heifers may produce slightly heavier calves at weaning than non-precocious [10], in addition, to be related with higher growth rates and better product quality [11]. 

Selecting for female sexual precocity involves reducing the age at first calving, which is a trait of easy and of a relatively early measurement, but usually presents low heritability [12]. An alternative is to consider early heifer pregnancy as an indicator trait of sexual precocity. This is a binary trait with moderate to high heritability defined based on age at first calving [13]. Age at puberty, which has been defined as the age at first corpus luteum (AGECL) obtained by regular ultrasound scanning of heifers [7,8,9,14,15], is also commonly used to evaluate female fertility and sexual precocity in tropical beef cattle. Due to the challenge of having to perform multiple scan examinations of heifers to derive AGECL accurately, Corbet et al. [16] proposed a single ultrasound examination of heifers at 600 days of age. The presence or absence of corpus luteum (CL) at 600 days, e.g., CL score, is more feasible to collect on a large scale [17]. According to Corbet et al. [16], the timing of 600 days concurs to the average age at puberty in tropically adapted composite breeds that occurs between 580 and 650 days. This is probably the moment when sire variation for the pubertal rate of their daughters would be utmost. It also coincides with the recording of other genetically evaluated traits, such as the ultrasound-scanned carcass characteristics. It is worth mentioning that, regardless of the indicator trait used, traditional selection for female sexual precocity is challenging since sires genetic evaluation requires recording their relatives, mainly dams and progeny, which limits annual rates of genetic progress.

In general, the lack of sexual precocity is a bottleneck for female cattle efficiency in Zebu cattle, directly impacting the herd profitability in tropical areas [18]. The age at puberty in Zebu cattle is around 25 months [19], and the age at first calving would range between 21 and 49 months with an average higher than 3 years [20]. These values highlight the importance of improving the sexual precocity of tropical cattle. The genetic improvement of heifer’s sexual precocity allows optimizing the productive cycle by reducing the number of unproductive animals in the herd. Here, we present and discuss the state of the art of genetic evaluation for sexual precocity in Zebu beef cattle, covering the definition of measurable traits, genetic parameter estimates, genomic analyses, and a case study of selection for sexual precocity in Nellore breeding programs.

## 2. The Genetics of Heifers Sexual Precocity

Female sexual precocity is related to the age at puberty, a polygenic trait regulated by a complex network of biochemical processes that culminates in the first ovulation generally followed by a short estrous cycle and subsequent regular cycles [21]. The age at which heifers reach puberty is highly influenced by environmental effects [18,22], but additive genetic effects are also important, especially in *Bos indicus* cattle [15,21]. Thus, together with adequate heifer development management, genetic selection could considerably reduce the age at puberty in tropical cattle [23].

### 2.1. Which Traits to Select?

In general, selection for sexual precocity aims to reduce the age at puberty and at first calving, with a consequent increase in the herd profitability. In this case, sexual precocity is an economic trait (selection objective) that could be expressed as various indicator traits (selection criteria) such as heifer pregnancy at about 14 months of age [24] or even as age at puberty, measured by the observation of the first corpus luteum [15], age at first calving [25], first service conception rate [26], and the number of antral follicles [27]. The target trait, e.g., sexual precocity, could be considered as both objective and criterion in the selection index, but not necessarily all traits in the selection criteria are included in the breeding goals [28]. Scrotal circumference, for instance, commonly used in beef cattle as an indicator trait of fertility and sexual precocity [25,29], is a good example of a selection criterion not included in the breeding objectives. Indeed, in livestock, it is common that some target traits, e.g., puberty, may be difficult or expensive to measure. Such economic traits have been traditionally improved by the correlated response to selection on easy-to-measure indicator traits that are considerably heritable and highly correlated with the breeding goals. 

In practice, an objective of selection is represented by a combination of economic and genetic values for a number of traits that influence the income and/or expenses of a specific production system [30]. In beef cattle, the breeding objectives usually include reproductive (with the sexual precocity being one of the most economically relevant traits), growth, and carcass quality traits [24]. Many other groups of economically important traits, e.g., feed efficiency, gas emissions, and meat quality, currently most studied in the context of the genomic selection approach, are still not normally included in the selection indexes [31,32]. With the focus on selecting for sexual precocity, phenotyping, and genetic knowledge involving this target trait, as well as information on its genetic correlation with other economic traits, are essential to design efficient selection strategies in beef cattle. 

#### 2.1.1. Puberty Traits

The direct selection for puberty requires measuring female age at puberty, which can be based on oestrus observation and progesterone concentration [15]. To obtain such measurements on a large scale would be challenging and expensive, limiting its adoption in commercial herds. The use of ultrasonography to measure ovarian activity, in particular, follicular size and the occurrence of a *corpus luteum* or albicans (CL or CA), as an alternative to determining heifer puberty, has been suggested in tropical cattle [15]. Nevertheless, scanning thousands of heifers approximately every 4–6 weeks to properly determine the appearance of the first CL would be not feasible for commercial herds [17]. Even adopting strategically timed ultrasound examinations [16], adaptations of management might be required with consequent increases in the production cost.

Despite the difficulty to directly measure puberty-related traits, heritability estimates of moderate to high magnitude have been reported for such traits in Zebu cattle raised under tropical conditions. Vargas et al. [33] reported, in Brahman, the heritability of 0.42 for age at puberty, defined as the age at first detected ovulatory estrus. Johnston et al. [15], using ultrasound to scan the ovarian activity of heifers, reported heritability estimates of 0.57 and 0.52 for age at first CL in Brahman and Tropical Composite, respectively. These authors also reported heritability estimates for weight, fat depth, and condition score at first CL, as well as for the presence of a CL or CA into first mating and on the scanning day into mating. The heritability of these traits ranged from 0.20 (presence of a CL or CA on the scanning day into mating) to 0.56 (weight at first CL) in Brahman and from 0.07 (presence of a CL or CA on the scanning day into mating) to 0.46 (weight at first CL) in Tropical Composite populations. In general, these estimates indicate that puberty traits, particularly the age and weight at first CL, might respond to selection in tropical beef cattle. 

Beyond heritability estimates, Johnston et al. [15] reported genetic associations between Brahman heifer puberty and production traits evaluated at the wet (ENDWET) and the dry (ENDDRY) seasons. Respectively for the ENDWET and ENDDRY measurements, these authors found that age at first CL is likely to be favorably genetically associated with fat depth P8 site (−0.35 and −0.33), scanned 12/13 rib fat (−0.29 and −0.38), body condition (−0.53 and −0.43), live weight (−0.33 and −0.20), and scanned eye muscle area (−0.36 and −0.22). Grossi et al. [34] reported favorable genetic correlations between age at first calving and females body weight-adjusted to 365 (−0.38) and to 450 days of age (−0.33). These results suggest that selection for increasing heifer growth, fatness, or condition score could improve heifer sexual precocity. It is worth mentioning, however, that the relationship between growth and fatness with puberty traits is not expected to be linear. Indeed, excess fatness or body weight would negatively impact age at puberty [18,35]. Little evidence of unfavorable genetic relationship between heifer puberty traits and carcass and beef quality has been reported [36]. On the other hand, the genetic antagonism observed between age at first CL and feedlot residual feed intake (–0.60) [15] highlights the importance of including both feed efficiency and puberty traits in the selection indexes. This would be especially relevant for breeding programs in the tropics focused on sustainable intensification of beef production, where sexual precocity and feed efficiency traits play an important role in herd profitability [18,37]. 

#### 2.1.2. Indicator Traits of Puberty

Scrotal circumference at different ages, age at first conception or at first calving, and heifer early pregnancy are among the most commonly used sexual precocity indicator traits in beef cattle [25,38]. These traits are measured more easily than puberty traits and can be part of the data routinely collected in the herds [39,40].

##### Scrotal Circumference (SC)

In beef cattle breeding programs, SC is routinely measured at young (e.g., yearling) ages to be used as a selection criterion for improving both male and female sexual precocity. Moderate to high (0.25 to 0.58) heritability estimates have been reported for SC [29,39,41,42,43,44,45,46]. In a meta-analysis study, Rojas de Oliveira et al. [47] reported heritability estimates of 0.43, 0.56, and 0.46 for SC at 365, 450, and 550 days, respectively, in Nellore cattle. The relationship between SC and heifer age at puberty is likely to be higher when SC is measured at younger ages. Johnston et al. [9], for instance, reported genetic associations of −0.21, −0.17, and −0.06 between, respectively, SC measured at 12, 18, and 24 months of age and age at puberty measured by the observation of the first *corpus luteum*. Favorable genetic correlation estimates between SC and age at first calving (−0.61 to −0.13) [20,29,39,41,43,48] and between SC and heifer early pregnancy (0.12 to 0.52) [29,42,48], indicate that the use of SC as a selection criterion can result in a decrease in age at first calving and a higher probability of precocious pregnancy or calving, increasing the herd sexual precocity.

##### Age at First Conception (AFCo) or Calving (AFC)

AFCo and AFC refer, respectively, to the age, in months or days, at the first positive pregnancy diagnosis and at the first calving. These traits are easier to measure than puberty traits but usually present low to moderate (0.07 to 0.24) heritability estimates [20,29,39,49,50,51,52,53]. Furthermore, part of the genetic variability of these traits is not captured because heifers are usually exposed to mating only when they reach a certain age or weight, in a breeding season with a predefined fixed period. This practice partially impairs the identification of sexually precocious females since they are exposed late to reproduction [42,54]. Another disadvantage for the genetic evaluation of AFC is that only females that have calved are considered in the analysis. 

AFCo and AFC are highly genetically correlated to each other (0.96) [39], suggesting that they are influenced by practically the same group of genes. A favorable genetic correlation between AFC and stayability [20,48,51,52,53,55] indicates that selection for sexual precocity can result in higher cow productivity. 

##### Heifer Early Pregnancy (HP)

HP is a binary trait expressed as success or failure of the heifer in conceiving or calving precociously, given a specific age. Unlike the AFC trait, HP is obtained for all heifers and normally presents moderate to high heritability. Heritability estimates for the probability of heifer conceiving at 16 months of age in Nellore range from 0.44 to 0.55 [13,29,56]. In addition, in Nellore, but for HP at 14 months of age, Van Melis et al. [57] found the heritability of 0.53. Defining HP as the probability of precocious calving at 24, 26, 28, and 30 months of age in Nellore, Bonamy et al. [58] reported heritability of 0.56, 0.50, 0.46, and 0.29, respectively. In general, these estimates indicate that HP should respond rapidly to direct selection, given that a higher response to selection is expected for heifer early pregnancy at younger ages. In general, HP is favorably genetically associated with AFC (−0.98 to −0.35) [49,55,59], stayability (0.09 to 0.73) [48,52,55,58], cumulative productivity (0.67), heifer rebreeding (0.83), and number of calves at 53 months of age (0.91) [55,59]. Focusing on selection for HP, the sustainable intensification of beef production in the tropics can be practiced considering two scenarios:

- Scenario 1

For Patterson et al. [22], optimizing the lifetime productivity would require first calving by 24 month of age, which implies that heifers must conceive until 15 mo of age. Some Nellore breeding programs started exposing heifers to mating at 14 months of age, and the probability of pregnancy at this age has been proposed as an indicator for sexual precocity [55,58,60]. Heifers calving at 2 years of age produced, on average, 0.7 more calves by the time they reach 6.5 years than those calving at 3 years [61] cited by [18]. Selecting for a higher probability of pregnancy at 14 mo would help to reduce the number of unproductive animals with a consequent increase in the number of weaned animals in the herds [58,62]. 

There are evidences that selection for early heifer pregnancy at younger ages would produce a rapid response, causing no negative impact in reproductive, growth, carcass, and feed efficiency indicator traits in tropical beef cattle [58]. It is important, however, to highlight that the nutritional requirements of precocious females tend to increase, especially at the time of the first rebreeding when they are still growing and nursing calves. Indeed, the relatively low heritability estimates for primiparous rebreeding, which would range from 0.03 to 0.18 [63,64,65], suggest an expressive environmental effect, e.g., nutrition and reproductive management, on primiparous rebreeding rates. Nevertheless, age at first calving is favorably genetically associated with heifer rebreeding [66]. Schatz et al. [67] showed that selection for fertility increases pregnancy rates of heifers exposed to reproduction at yearling in tropical conditions. In order to achieve reproductive efficiency, it is key to find the proper balance between sexual precocity and weight gain selection [68].

- Scenario 2

Common reproductive management adopted by breeding programs in Brazil is to consider two breeding seasons: (1) fall season—females with approximately 15 to 17 months of age are exposed to mating for 60 days, in an attempt to identify sexually precocious females; (2) summer season—all females are allowed to breed for a period of 70–90 days. In this scenario, heifers that fail to conceive in the fall season (non-precocious) have a second opportunity in the summer season, and precocious heifers have the advantage of having a longer period from their first calving to conception (10 to 11 months) than non-precocious (3 to 5 months). Females (dams and heifers) that fail to conceive in the summer season are culled [10,11,69]. This management practice favors the achievement of higher percentages of early pregnancy and heifer rebreeding rates in the herds. Additionally, anticipating heifer reproductive life was found to be favorably associated with cow cumulative productivity and longevity in tropical beef cattle [11].

As well as for sexual precocity, improving the probability of a cow to remain productive in the herd for a specific period of time (stayability) has a great impact on the herd profitability [24]. Indeed, stayability can be up to 4.10 times more economically important than heifers sexual precocity [62]. In this sense, the reproductive practice of having an early breeding season for identifying precocious heifers also impacts the expression of stayability. As reported by Terakado et al. [10] and Fernandes Júnior et al. [11], in scenario 2, precocious females presented a higher probability of staying in the herd for a longer time (success stayability) compared with non-precocious females. This might be related to the fact that precocious heifers have more time for recovering from their first calving to their first rebreeding than non-precocious [10]. In addition, with a longer period of days open, precocious females have more time for post-partum recovery, could conceive earlier in the subsequent breeding season, and are likely to wean heavier calves (due to its occurrence in the wet season). All these events are expected to increase the probability of early pregnancies in subsequent breeding seasons, favoring the productivity of the whole production system. 

#### 2.1.3. Sexual Precocity Evaluation Using High-Throughput Technologies

High-throughput phenotype technologies generate accurate real-time animal-level information, allowing to evaluate the dynamic nature of several traits, which might be used to enhance the genetic improvement of sexual precocity of heifers. In this context, digital image analysis and computer vision systems (CVS) are promising tools for applications in animal science [70]. These technologies, related to the process of extracting and interpreting information from images, can be an alternative to monitor animal growth in real-time with minimal animal stress and much lower cost and labor. Constant measurement of traits through high-throughput imaging can reduce management costs, optimize decision-making in livestock operations, and open new possibilities in selective breeding [70]. The application of such technologies has already been extended to biometric measurements [71] in beef cattle as well as production traits such as body weight and average daily gain [72] and body condition score [73]. 

Females’ sexual precocity is a complex trait highly influenced by heifer development, which is characterized by several phenotypic changes. Since traditionally, it is recommended that heifers reach 60 to 65% of their adult body weight at the beginning of the breeding season [18,74], such information could be elucidated by monitoring repeated measures of body weight and body condition continuously over time or at discrete intervals during the growth phase. Additionally, infrared thermographic cameras can be used to detect estrus in cows, providing an indication of when ovulation is most likely to happen [75]. 

Some growth curve parameters were shown to be good predictors of sexual precocity. For instance, Oliveira et al. [76] and Gaviolli et al. [77] reported negative and high genetic correlations between maturation rate (*k*) of growth curve models and AFC for Guzera (−0.67) and Canchim (rg = −0.83) cattle, and Inoue et al. [78] reported a high correlation between *k* and conception rates (0.91) in Japanese Black cattle. These results suggest that the selection for a higher maturation rate will positively impact the age at first calving and conception rates. Using high-throughput phenotyping technologies, traits such as weight at different ages (growth curves) and body condition could be more easily and accurately evaluated and used to assess heifer sexual precocity in large-scale production systems. 

### 2.2. Genomic Selection 

An alternative to increase the genetic progress of sexual precocity is the adoption of genomic selection (GS). GS refers to the evaluation of animals based on direct genomic values derived from the sum of the estimated effects of single nucleotide polymorphism (SNP) genotypes of selection candidates [79]. When implemented based on traditional SNP arrays, GS relies on the existence of linkage disequilibrium (LD) between SNPs and quantitative trait loci (QTL) [79]. Dense SNP panels covering the entire genome are used under the expectation that all QTL are in LD with at least one SNP marker [31]. Using whole-genome sequencing (WGS) data, predictions no longer have to rely on LD since, theoretically, phenotype causal mutations are directly included in the analysis [80,81]. 

Basically, the implementation of GS for a specific trait, e.g., heifer’s sexual precocity, requires an equation that predicts breeding value from SNP genotypes [32]. This equation has to be constructed from a large number of individuals measured for the trait and genotyped for the genome-wide markers, the so-called reference population [32]. Thus, QTL genetic effects are captured by regressions of phenotype on SNP genotypes in order to estimate the effects of each marker. The prediction equation, combining all the marker genotypes with their respective effects, is then applied to a target population, which is a group of candidates to selection with genotypic but no phenotypic information [32]. The definition of which model and pseudo-phenotype should be used in a specific GS implementation is normally made based on model comparisons in terms of prediction accuracies [31,79,80,81,82]. Zhang et al. [8] reported genomic prediction accuracy of 0.35 (Brahman) and 0.23 (Tropical Composite) for AGECL. These authors stated that these relatively low accuracies of predictions reflected the limited number of data available (996 Brahman and 1097 Tropical Composite), associated with greater genetic complexity mainly in the Tropical Composite. In general, beef cattle populations consist mainly of various breeds and/or a mixture of breeds, making it difficult to apply GS in this field [31]. Genomic prediction in tropical beef cattle in scenarios with a small proportion of breeds in the reference population benefits from the use of multi-breed genomic evaluations [17].

In Nellore cattle, accuracies ranging from 0.23 to 0.63 were reported for age first calving [83,84] and from 0.25 to 0.67 for heifer early pregnancy, respectively [84,85]. Evaluations of age at puberty in Brahman and Tropical Composite indicated prediction accuracies varying between 0.20 and 0.50 [8,17,86]. Differences in terms of reference population size, study design, population structure, among others, explain this high predictive ability variation among studies. However, it is possible to infer that genomic prediction accuracies for heifer sexual precocity in tropically adapted beef cattle range from low to moderate. 

Genomic information becomes especially relevant to accurately select young sires for traits that are difficult or expensive to measure and/or those that are measured late in an animal’s life or only measured on females [31,81,87]. To account for Mendelian sampling effects in the prediction of genetic merit of an individual, traditional mixed models rely on phenotypic recording on that individual or on its relatives [88]. With only ancestral records, animal genetic merit basically reflects the parent average effects, which limits the accuracy of prediction and also the chance of identifying young bulls with genetic merit superior to existing selected sires [89]. Considering, for instance, that the heritability of age at first calving ranges between 0.08 and 0.21 [84], the breeding value accuracy of a young bull evaluated based only on the information of his dam is expected to range between 0.14 and 0.23. In such a scenario, records on progeny or other relatives are necessary in order to increase the accuracy of selection, increasing the generation interval [89] and costs of the breeding program. On the other hand, by estimating the genetic merit of animals at a very young age with higher accuracy in comparison to traditional pedigree-based methods, GS allows drastically reducing the generation interval [81,87]. In this sense, accurate genomic predictions for heifer’s sexual precocity would allow the selection of genetically superior young bulls, which might accelerate genetic gain [8,17]. 

Recently, Warburton et al. [86] showed that genomic predictions for age at puberty in tropically-adapted beef heifers could benefit from the use of WGS data. The general idea is to add preselected WGS variants using genome-wide association study to a regular SNP array [90]. Reasonable improvements in accuracies were achieved by adding a relatively small number of highly relevant WGS variants to low-density panels when using Genomic Best Linear Unbiased Prediction (GBLUP) as the prediction model [86]. Although, in general, high-density panels may be sufficient for accurate genomic predictions, studies have indicated that adding preselected sequenced variants to these arrays may also increase predictions for several traits, including fertility [90].

### 2.3. Genotype by Environment Interaction (G × E) for Sexual Precocity in Breeding Programs

G × E basically refers to the change in variation and/or in the ranking of genotypes evaluated in different environments. G × E represents an important source of sexual precocity variation in beef cattle raised in tropical environments [23,85,91], which are characterized by a wide range of animal production situations, including areas with more favorable conditions than others. In the presence of G × E, environmental sensitivity might increase with selection for high phenotypic value in continuously improving environmental conditions [92]. 

**Figure 1:**
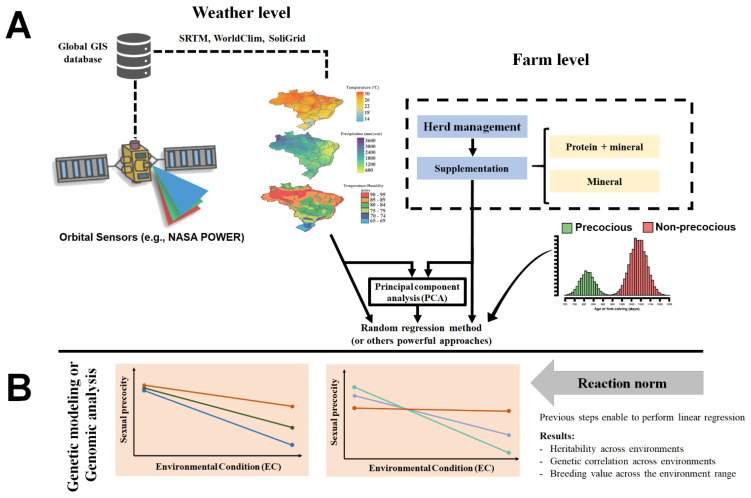
Schematic conceptualization for the evaluating of genotype by environment interaction in beef cattle raised in tropical regions. (**A**) An example of environmental gradient derived based on the combination of geographic information systems (GIS) and herd-level management. (**B**) Graphical representation of linear reaction norm evaluation of the response of breeding values to environmental changes.

Evaluation of animal’s response to environmental changes has been widely studied using reaction norms (RN) [92,93]. Such models use random regression curves with trajectories determined by continuous environmental descriptors to describe the response of each individual to environmental variation [94]. Traditionally, in animal breeding, these descriptors are based on contemporary group solutions for a specific phenotypic variable, e.g., post-weaning weight gain (PWG) [85,94,95]. According to [94], PWG was an adequate trait to assess animal sensitivity to environmental changes since after weaning, animals are normally exposed to a wide range of production conditions, being especially true in tropical pasture-based systems. For Rellstab et al. [96], a properly environmental characterization was a fundamental step to understanding how the environment regulates the phenotypic variation of genotypes under diverse conditions. Hence, the authors suggest using a combination of herd-level environmental conditions and weather data from geoprocessing technologies into G × E evaluations (Figure 1) to increase the prediction accuracies of statistical models. In this sense, environmental information from different sources could be condensed by principal component analysis (Figure 1) and used in G × E evaluation models [96].

As beef cattle are raised in highly heterogeneous environmental conditions in tropical production systems, heterogeneity might exist in the (co)variance components for economically relevant traits across environments [97]. For sexual precocity traits, results have shown that G × E may affect the genetic variance and animal ranking [85,98,99,100], suggesting that breeding programs must consider this effect in their selection decisions. One alternative would be to select for reduced environmental variance in order to achieve a more homogeneous production across different environmental conditions [101]. Reducing animal sensitivity to environmental influences would, for instance, minimize the risk of increasing the number of non-precocious heifers’ categories in the herd.

Modeling the effect of G × E allows selecting animals based on their resilience or robustness. The selection based on resilience aspects aims to improve animals’ capacity for dealing with adverse environmental conditions [102,103,104]. In the context of sexual precocity, selecting for lower sensitivity to environmental variation, i.e., higher resilience (slope of reaction norm equal to zero), will lead to animals with less variation for sexual precocity indicator traits across environmental conditions. Berghof et al. [105] indicated that animal resilience could be addressed by the skewness of deviations, environmental variation, the autocorrelation of deviations, or the slope of a reaction norm. These indicators allow estimating breeding values with less variation to stressors factors, given that a general resilience is expected when these values are close to zero. In tropical areas, where animals are frequently exposed to a wide range of environmental conditions, these indicators could be used to identify sires producing heifers that exhibit a remarkable sexual precocity across different beef cattle production systems.

Selecting for robustness, in turn, would improve sexual precocity combined with resilience to stressor factors, allowing a high expression of this trait in a wide variety of environmental conditions [106]. Improving robustness might increase the proportion of heifers that are able to grow and keep functional metabolism to express sexual precocity in harsh environments. In addition, when environments become less restrictive, these heifers tend to show higher genetic merit for sexual precocity than non-precocious heifers, which tend to be less adapted to environmental changes [23,85]. Being related to animal performance and welfare, selection for sexual precocity and robustness would potentially influence profitability in sustainable beef production systems.

#### G × E Interaction for Sexual Precocity in Genomic Era 

The G × E is a mostly tropical issue that must be addressed in this region. Despite its importance in animal breeding by frequently causing a reduction in response to selection [107], G × E is often ignored in the genetic evaluations. Perhaps the biggest obstacle to modeling G × E in the tropics is how to make these results available and use them at the level of breeders and producers. Moreover, this effect tends to increase statistical modeling complexity, and, mainly when evaluations rely only on pedigree-based analysis, there is often a lack of prediction accuracies, particularly in extreme environments [108]. In this sense, incorporating genotypic information to reaction norm models increases environment-specific breeding value accuracies [108] and has extended the evaluations from the individual to the SNP level, allowing to infer SNP effects along the environmental gradient [94]. Compared to pedigree-based evaluations, genomic reaction models have provided higher prediction accuracies for economically relevant traits, including sexual precocity [85,109,110,111]. In the genomic era, large-scale allelic information replicated across environments is available, which can partially overcome the main problem in traditional G × E analysis that is a frequently reduced degree of sires having offspring in various environments [112]. Thus, genomic selection breeding programs accounting for G × E represent promising tools for improving production efficiency in livestock [109].

The performance of tropically-adapted beef cattle is likely to be affected by G × E interactions. Carvalheiro et al. [94], working with post-weaning weight gain in Nellore cattle, studied the sensitivity to environmental variation at both the individual and molecular level and found evidence that they are not linear along the environmental gradient. Still, according to Carvalheiro et al. [94], the genomic regions affecting sensitivity in harsher and less challenging environments were not the same, whereas some of the genes within those regions participate in common biological pathways linked to adaptability. Using genomic reaction norm models in genetic evaluations of sexual precocity-related traits in Nellore cattle, Mota et al. [85] reported evidence of significative G × E interaction for early pregnancy and scrotal circumference traits with changes in SNP effects, genetic variance estimates, and breeding values sensitivity along the environmental gradient. 

Differences in environmental conditions, mainly related to nutritional aspects, affect cattle’s sexual precocity by influencing metabolic status through changes in glucose, insulin, and reproductive hormones [98,113,114]. All these factors would be directly involved with specific metabolites with a striking effect on oocyte development, ovulation, embryo growth and survival, and pregnancy rate [91,115], indicating that heifer sexual precocity is an energy-dependent process. Indeed, genome-wide association results for sexual precocity across environmental conditions identified genes involved with the regulation of energy expenditure in tropical beef cattle [91]. These last authors reported that regions linked to metabolic homeostasis were associated with AFC in restricted environments, whilst those related to the energy and lipid metabolism were associated in the intermediate environment level. Moreover, they found that genes involved with metabolic substrates play a role in the expression of sexual precocity in the most favorable conditions. 

A better understanding of molecular mechanisms underlying G × E effects on sexual precocity in tropical beef production systems is important to design efficient selection strategies to improve puberty in stressful conditions. Probably, the accuracy of selection for heifer sexual precocity will be improved with statistical models accounting for genomic regions presenting environment-dependent sensitivity. Additionally, modeling G × E is necessary to enable international reference populations for genomic selection across environments and countries [112]. 

### 2.4. Molecular Genetics

A better understanding of the genetic control of puberty-related traits may contribute to designing improved selection strategies for sexual precocity. With this purpose, genome-wide association studies (GWAS) were developed in order to identify candidate genomic regions or genes with major effects on the phenotypic expression of traits of interest. To date, a total of 108 QTLs were described at the release 45 of the QTLdatabase website [116] for the trait female sexual precocity. For other age puberty-related traits such as age at first breeding, age at first *corpus luteum*, age at first insemination, age at first service, age at puberty (male and female), and early puberty in cattle, a total of 10,623 QTL were reported. 

In Table 1, summarized are the most recent GWAS results for heifer’s sexual precocity traits in *Bos indicus* and tropical composite breeds. In general, results from these association analyses (Table 1) have confirmed the complex nature of sexual precocity traits, which are generally affected by a large number of QTL with small effects, even though only candidate regions or genes with the highest effects are normally discussed in the manuscripts.

GWAS for sexual precocity in Nellore heifers, for instance, have suggested various candidate regions and genes such as the *Kiss-1*, *PAPP-A2*, *ESRRG*, *PAPP-A*, *MBL-1*, *and XKR4*, which are related to GnRH release, estrogen, and pregnancy pathways, affecting pubertal related traits [129,130,131]. The *ESRRG*, located on BTA16 at 19 Mb (ARS-UCD1.2), also plays an important role in lipid metabolism, regulating fatty acid and glucose levels [132,133], and in thermoregulatory responses [134]. The *XKR4* (BTA14, 22 Mb) was associated with climate adaptation in *Bos indicus* cattle [135], fat deposition [136,137], feed intake, and growth [138]. Such possible pleiotropic effects, indicated by regions or candidate genes influencing the expression of more than one trait, have been frequently reported in GWAS [139] and corroborate, in general, with genetic correlations estimated for different economic traits [140]. 

The region located on BTA14 at 20–28 Mb (UMD3.1) is one of the most known large-effect pleiotropic regions affecting the expression of several economic traits, including those related to sexual precocity across different breeds (Table 1). This region harbors the Pleomorphic adenoma gene 1 (*PLAG1*) that is a transcription factor encoding the zinc finger protein PLAG1. Strong GWAS signals at this genomic region for a number of traits in cattle, including heifer puberty, are generally attributed to a causative mutation present in *PLAG1* [130,141], but other genes located in the same region of BTA14 (such as the *TMEM68*, *NPBWR1*, and *OPRK1*) were also associated with reproductive performance in different species of mammals [91,142,143,144]. The *TMEM68* and *OPRK1* are related to neurons development, associated with the glial cell, a key component to promote responses to growth factors and GnRH [91]. In addition, the *TMEM68* gene was significantly associated with residual feed intake and average daily gain in crossbred steers [138].

As pointed out by Fernandes Júnior et al. [145], an important consideration about GWAS results for complex traits is that the candidate regions identified in different populations are frequently different across studies, even though considering similar traits and breeds. According to Ma et al. [146], this difficulty of finding major QTL shared across studies is especially true for fertility traits, which could be related to the complex nature of these traits, associated with the fact that they usually presents low heritability estimates and are often highly influenced by management decisions. In this sense, a meta-analysis of GWAS across breeds appears as an alternative to validate genomic regions and increase the QTL detection power, including pleiotropic regions [126,141,147]. Based on meta-analysis results, Melo et al. [127] were able to validate 62 SNPs and 30 candidate genes associated with puberty traits across tropically-adapted beef cattle breeds (Brahman, Nellore, and Tropical Composite cattle) and Tahir et al. [148] reported 1359 significant SNPs in a meta-analysis of pregnancy at first mating opportunity, first conception score, and rebreeding score in Brahman cattle. These results suggest that large-scale meta-analysis would allow a more accurate discovering of QTL affecting fertility and sexual precocity than a single GWAS.

Candidate genes, identified by GWAS or meta-analysis, are generally used in enrichment analysis in order to identify over-represented pathways underlying complex traits. This is important for a better understanding of the genetic mechanisms involved with the expression of complex phenotypes. Basically, in pathway-based association analysis, candidate genes are assigned to biological processes based on their function and properties of their encoded products, and then a statistical test, e.g., Fisher’s exact test, is used to identify a possible overrepresentation of the list of candidate genes used as input in a given pathway [149]. For early puberty related traits, gene set enrichment analyses have pointed to biological processes related to response to hormone levels as estrogen, estradiol, insulin, GH, as well neuron development, pregnancy, glial cell proliferation, ovulation cycle [91], immune system, apoptotic process, response to stimulus [122] among others. This variety of biological processes significantly associated with the expression of sexual precocity in beef cattle reinforces the complexity around the regulatory system of heifer puberty. 

In general, the combination of GWAS, meta-analysis, and functional pathway enrichment analysis have provided great advances in the understanding of genetic control of pubertal-related traits in tropically-adapted beef cattle [148]. Such knowledge may directly contribute to improving selection strategies for sexual precocity in tropical cattle. As reported by MacLeod et al. [150], prediction accuracies of genetic merit for complex traits might increase by incorporating prior biological information in the analysis. Furthermore, GWAS using whole-genome sequencing data may lead to the identification of causal variants affecting fertility traits, such as the *SMC2* mutation that causes embryonic loss [151]. Such variants can greatly increase the accuracy of selection for sexual precocity. 

When variants are found in gene regions, especially in exons, determining the cause and effect of these variations is a crucial issue. For instance, exome sequencing made it possible to identify causal mutations in underlying defective bovine embryo development [152] and fertility-associated haplotypes [153]. In this sense, large-scale data sets from different “omic sciences” such as transcriptomics, proteomics, metabolomics, metagenomics, phenomics, etc., have allowed the emergence of a systems approach to improve the understanding of biological processes and could collaborate for more sustainable animal production in the tropics.

In the tropical environment, the effect of heat stress, food, and water scarcity during summer affects reproduction performance in most livestock species. Low reproductive hormones secretion, the incidence of abortion, and embryonic death are among the problems compromising reproductive efficiency [154]. Investigating the transcriptome and/or proteome has sought to understand how the expression of genes and proteins, respectively, are affected by tropical environments and how this information can be combined with the use of reproductive tools for sustainable livestock production in a tropical environment.

Rocha et al. [155] used the transcriptomic approach (RNA-Sequencing) to identify the gene expression profile in leukocytes from 18 days timed artificial insemination between pregnant and non-pregnant Nellore heifers. The presence of a viable conceptus stimulated expressive differentially expressed genes in leukocytes. The discovery of these early-pregnancy hub genes on immune cells retrieved from peripheral blood showed insights about the development of methods to help predict positive pregnancies earlier. 

In Brahman heifers, transcriptomic studies showed a large list of differentially expressed genes (genes already considered to be markers and also new genes) related to puberty [130,156]. Genes with functions in similar biological processes and pathways, such as those related to development, cellular maintenance, immune, and lipid systems. Based on these findings, it is possible to infer that functional activity for puberty can be modulated for many different genes that act in similar systems according to genetic factors and environmental conditions available.

Using advanced proteomic technologies, Nguyen et al. [157] and Tahir et al. [158] identified differentially expressed proteins, their functions, and interactions in Brahman heifers. These authors showed that the puberty and fertility of these animals depended on proteins related to steroid synthesis and ovarian signaling pathways. These results consolidate the information that has already been obtained with GWAS and transcriptome results, which show causal variations and transcriptional profiles related to these same mechanisms in Brahman cattle.

Metabolomics is another emergent “omics” area that has provided a deeper knowledge of the role of metabolites in reproduction, such as the metabolomic signature of the ovarian follicular fluid and embryos development. These results could improve natural and assisted reproduction in bovine [159]. The relationship between body condition, metabolic status, liver function, and reproduction has been studied extensively in periparturient and early lactation dairy cows of taurine origin [160,161]. 

Sundrum et al. [161] discussed the implications of metabolic stress caused by changes and disturbances in nutrition at a metabolic level in dairy cows. In accordance with these authors, dairy cows are more easily successful in adapting and preventing dysfunctional processes in the transition period, when the gap between nutrient and energy demands and their supply is restricted. The pathophysiology of metabolic disorders and postpartum diseases is multifactorial and often driven by several interlinked risk factors. Metabolomics could help to better understand these metabolic processes, but such studies evaluating reproductive traits and postpartum diseases, especially puberty, in tropical cattle still remain limited.

The big data generated by the omics approach and the joint studies with different levels of information, called multi-omics integration, have been used to investigate the dynamics of complex traits. Cánovas et al. [162] and Fonseca et al. [163], for instance, combined results from GWAS and RNA-Seq to build co-expression gene networks showing which genes and how their complex interactions were related with puberty in Brangus heifers (Brahman × Angus). Fortes et al. [164] used integrative analyses of transcriptomics and proteomics from Brahman heifer’s uterus tissue, finding new protein biomarkers for puberty. Most of the time, these biomarkers are pleiotropic key regulators. Genes with pleiotropic effects related to crucial biological processes that regulate economically relevant traits associated with fertility, production, and health, could be used to assist the selection of superior animals for these traits.

The biochemical and molecular knowledge of complex traits in cattle raised in the tropics, combined with sustainable environmental management, would lead to new study proposals in the area of systems biology. In addition, the knowledge acquired using functional approaches leads to a better understanding of the biological mechanisms at all levels. Elucidating interconnections involving the different network members (genes, RNAs, proteins, metabolites) is essential for the comprehension of the organism as a whole.

### 2.5. Case Study: Selection for Sexual Precocity in Nellore Breeding Programs

Nellore is a zebu breed, introduced in Brazil from India at the end of the XIX and the beginning of the XX century. The last importation was in the 1960s. In total, around 6200 animals were imported. Due to its adaptation to tropical conditions, including resistance to heat stress and endo and ectoparasites, in special ticks, this breed has spread around Brazilian tropical regions. Today, 80% of the beef cattle population in Brazil is composed of Nellore and its crosses [165]. 

In the 1980s, genetic evaluation programs were implemented by independent groups, starting to use EPDs and oriented mating systems [166], accelerating genetic progress. Nowadays, there are more than 10 private Nellore breeding programs, which together control roughly 700,000 cows per year. Since 1998, part of these programs joined their databases, auto denominated “Aliança Nelore” (Nellore Alliance), in order to develop research and publish sire summaries. These databases have pedigree and phenotypic records for several economically important traits of about 2.5 million animals.

**Figure 2:**
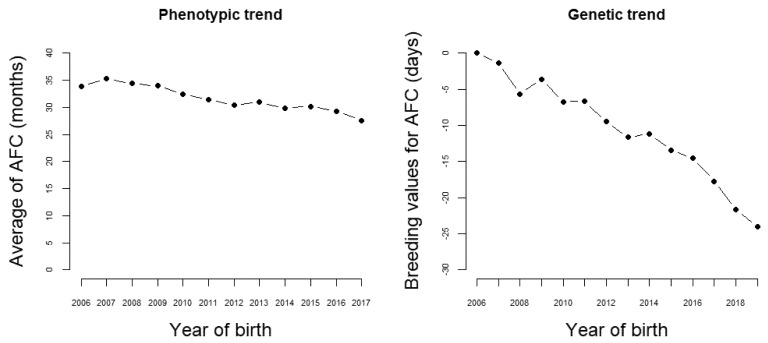
Phenotypic and genetic trend for age at first calving.

The results presented in this section are from one of the breeding programs composing the Nellore Alliance, chosen because it imposes a strong selection intensity on female sexual precocity. Presently, in this program, about 90,000 cows from 90 herds are annually controlled. In 12 years, the age at first calving phenotypic (AFC) means showed a strong decreasing trend, changing from nearly 34 to less than 28 months (Figure 2). Naturally, this change has happened partially due to improvements in management and nutritional conditions. However, the estimated genetic trend for AFC was of almost −2 days/year (Figure 2).

The phenotypic and genetic progress, verified in this period, was possible due to the so-called “precocious challenge.” Historically, in the breeding programs, females used to be first exposed to reproduction after achieving minimum weight and/or age, around 24 months. In the 1990s, in a few breeding programs, young females (16–18 months of age) started to be exposed in an earlier breeding season aiming to identify the “precocious” one. In this system, non-pregnant females at the end of this breeding season are given another chance to conceive in the regular breeding season, when all females are exposed. For several years, rates of pregnancy in the earlier breeding season were around 10–18% [10,167,168,169]. Lately, females are being exposed to reproduction at 12–14 months of age and, those that are pregnant at this age are considered “super precocious”. 

**Figure 3:**
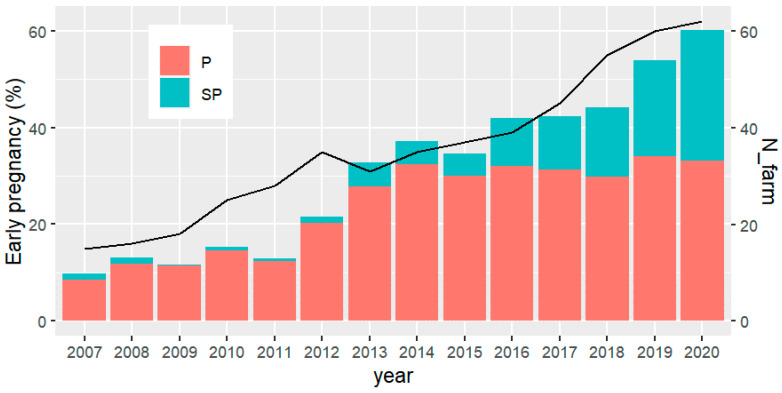
Number of farms (N_farm) implementing the so-called “precocious challenge” (solid line) and percentage of precocious (P) and super-precocious (SP) heifers (bars), according to the year of birth of the first product.

Results from the specific breeding program presented here show that the number of herds exposing young females to reproduction expressively increased from 2007 (~15) to 2020 (62). Likewise, the percentage of early pregnancy, including precocious and super precocious females, changed from around 10% to more than 60% in the same period (Figure 3). Important to note that from 2013 occurred an acceleration in both, number of herds and the percentage of early pregnancy. More recently, it has been found that with genomics, there is an increase in young sires EPD accuracies of about 50% [1]. Therefore, from 2017 the sire selection index has been modified to include AFC instead of scrotal circumference, selecting directly for female sexual precocity.

Economic advantages of decreasing age at first calving from 36 to 24 months of age are well documented [170,171]. In Nellore cattle, Terakado et al. [10] found that precocious females (conceiving around 16–18 months of age) had 33% and 28% higher chances to remain in the herd until 5–6 and 7 years of age, respectively, compared to cows conceiving at 24–26 months of age. Moreover, during their productive life, precocious females, on average, weaned 1.410 kg heavier calves than those non-precocious, with no differences in cow adult weight between the two groups. Although precocious females showed a greater calf mortality rate in the first calving, there was no difference in calf mortality between precocious and non-precocious females in subsequent calvings. In Nellore, the consequences of the “super precocious” system on female productive life are to be studied. A word of caution is necessary about this system regarding the need for improvement in young females’ management and nutrition. It is critical to consider that Nellore is generally raised on pasture, and selecting animals in more intensive production systems can lead to losses in adaptation. 

For Capper [172], the key to decreasing beef cattle production’s environmental impact is to improve productivity. To this author, the main aspects of improving herd productivity are reproductive efficiency, age at first calving or service (bulls), replacement and mortality rate. Reducing AFC decreases the number of non-productive animals in the herd and the production cycle, allowing better use of resources, with an impact on environmental sustainability. 

## 3. Conclusions

In general, puberty-related traits would quickly respond to selection by presenting moderate to high heritability estimates. In this context, the heifer early pregnancy (HP) trait, which expresses the success or failure of a heifer in conceiving or calving precociously at a specific age, is a promising selection criterion for improving sexual precocity in beef cattle. As a future perspective, the use of high-throughput phenotyping strategies in the evaluation of sexual precocity has been emerging with advances in digital image analysis and computer vision systems.

Some Nellore breeding programs have achieved a considerable decrease in both phenotypic and genetic trends for age at first calving by exposing heifers to mating at 14 months of age in order to identify and select precocious females. Consequently, the proportion of early pregnancy in these herds has increased from around 10% to more than 60%. The genetic improvement of heifer’s sexual precocity contributes to reducing the number of unproductive animals in the herds and promotes sustainability through better use of resources. 

Genome-wide association studies have pointed many quantitative trait loci affecting puberty traits in tropical cattle, and multi-omics approach, still at the beginning in livestock, has already presented promising results in multiple levels (transcriptomics, proteomics, metabolomics, metagenomics, phenomics, and so on). These molecular approaches have contributed to uncover specific molecular information, e.g., SNPs, genes, and biological pathways, with major effects on heifer’s sexual precocity. Modeling such information in genomic selection evaluation may increase prediction accuracies. Additionally, with whole-genome sequencing data, phenotype causal mutations could be identified and directly included in the analysis. Genomic selection breeding programs accounting for genotype by environment interaction also represent promising tools for accelerating genetic progress for sexual precocity in tropical beef cattle. 

## Figures and Tables

**Table 1:** Genomic regions associated with sexual precocity in *Bos indicus* and influenced *Bos indicus* heifers.

Phenotype	BTA	Pos (Mb) ^a^	Breed	Number of Phenotypes/Genotypes	Reference
First Service Conception	1	135.38	Brangus	861/802	[117] Fortes et al., 2012
	5	56.67			
	5	70.26			
	9	82.42			
	11	95.64			
Age at first *corpus luteum*	14	20–33	Brahman	837	[118] Hawken et al., 2012
	5	93–96	Tropical Composite	860	
Age at first *corpus luteum*	14	25.01	Brahman	1007	[119] Fortes et al., 2013
			Tropical Composite	1111	
First Service Conception	3	101.0–101.9	Brangus	830/796	[120] Peters et al., 2013
	8	25.0–26.9			
	15	69.0–69.9			
	16	43.1–43.9			
	19	49.0–49.9			
	24	53.0–53.9			
	26	8.0–8.9			
	26	16.0–16.9			
	27	33.0–33.9			
	29	22.0–22.9			
	X	108.1–108.9			
Heifer Pregnancy	2	41.0–41.9	Brangus	830/796	[120] Peters et al., 2013
	4	4.0–4.9			
	8	0.3–0.9			
	10	91.0–91.9			
	13	83.0–83.9			
	20	70.0–70.9			
Early Pregnancy	5	8.8–10.12	Nellore	73,359/1770	[40] Irano et al., 2016
	5	16.06–17.12			
	6	10.64–11.66			
	7	3.12–3.85			
	7	41.28–42.03			
	14	22.61–23.39			
	18	4.26–4.91			
	21	0.01–3.02			
	21	61.92–62.53			
	27	0.99–1.57			
Early Puberty	5	78.64	Nellore	55	[121] Nascimento et al., 2016
	6	59.02			
	9	8.44			
	10	33.82			
	22	10.41			
Age at first calving	4	17.46	Canchim	267,002/392	[122] Buzanskas et al., 2017
	4	98.31			
	27	35.19–35.21			
Age at first calving	2	6.17–7.17	Nellore	762/2992	[123] Mota et al., 2017
	8	106.27–107.27			
	9	40.97–46.61			
	14	16.54–17.53			
	14	20.39–24.67			
	14	26.20–28.84			
	14	31.25–36.95			
	16	43.94–44.93			
	16	68.23–69.23			
	17	57.29–58.28			
Early Pregnancy	5	72.52–74.46	Nellore	2283/2283	[124] Oliveira Junior et al., 2017
	5	76.52–78.48			
	5	80.63–82.46			
	14	22.50–24.48			
	14	28.56–30.48			
	18	54.51–56.45			
Number of antral follicles	2	122.53–124.48	Nellore	1099/2283	[124] Oliveira Junior et al., 2017
	8	6.51–8.47			
	11	69.52–71.47			
	14	22.50–24.48			
	15	8.50–10.46			
	16	70.50–72.45			
	22	14.50–16.47			
Age at first *corpus luteum*	7	23	Brahman	914	[125] Fortes et al., 2018
	21	23			
	19	49	Tropical Composite	798	
Meta-analysis for fertility traits	1	118.6	Nellore	AFC 1796/1796	[126] Melo et al., 2018
	2	95.917			
	3	49.43			
	4	110.44			
	6	118.43			
	7	94.71		EP (%) 1849/1849	
	8	68.3			
	9	75.61			
	10	16.76			
	11	104.93			
	13	16.09	Brahman	AGECL 1007	
	14	24.71			
	15	9.06			
	16	1.92			
	21	11.43			
	24	2.27			
	26	23.4			
	27	31.92			
	29	9.17			
Age at first *corpus luteum*	1	43.45	Tropical Composite	1097	[127] Melo et al., 2019
	14	25.24			
	15	9.06			
	16	24.328			
	21	6.83			
	23	27.78			
	26	23.4			
	29	9.17			
Heifer pregnancy	5	70.5–72.7	Nellore	1337/1337	[128] Oliveira Jr., 2019
	14	20.7–24.6			
Age at first calving	1	22.86–23.03	Nellore	185,356/3760	[91] Mota et al., 2020
	2	105.03–105.38			
	3	21.19–21.22			
	3	8.34–8.41			
	5	9.47–10.87			
	6	19.49–19.54			
	14	24.82–25.10			
	15	35.34–35.64			
	17	49.64–49.83			
	18	3.08–4.89			
	27	31.64–31.97			

^a^ All cited publications used the UMD 3.1 as reference genome assembly.

## Data Availability

Not applicable.
